# Achieving development goals for HIV, tuberculosis and malaria in sub-Saharan Africa through integrated antenatal care: barriers and challenges

**DOI:** 10.1186/s12916-016-0753-9

**Published:** 2016-12-12

**Authors:** Freya J. I. Fowkes, Bridget L. Draper, Margaret Hellard, Mark Stoové

**Affiliations:** 1 Macfarlane Burnet Institute of Medical Research, Melbourne, Australia; 2 Department of Epidemiology and Preventive Medicine, Monash University, Melbourne, Australia; 3 Department of Infectious Diseases, Monash University, Melbourne, Australia; 4 Centre for Epidemiology and Biostatistics, University of Melbourne, Melbourne, Australia

**Keywords:** Antenatal care, HIV, Tuberculosis, Malaria, Integrated services

## Abstract

**Background:**

The global health community is currently transitioning from the Millennium Development Goals (MDGs) to the Sustainable Development Goals (SDGs). Unfortunately, progress towards maternal, newborn and infant health MDGs has lagged significantly behind other key health goals, demanding a renewed global effort in this key health area. The World Health Organization and other institutions heralded integrated antenatal care (ANC) as the best way to address the inter-related health issues of HIV, tuberculosis (TB) and malaria in the high risk groups of pregnant women and infants; integrated ANC services also offer a mechanism to address slow progress towards improved maternal health.

**Discussion:**

There is remarkably limited evidence on best practice approaches of program implementation, acceptability and effectiveness for integrated ANC models targeting multiple diseases. Here, we discuss current integrated ANC global guidelines and the limited literature describing integrated ANC implementation and evidence for their role in addressing HIV, malaria and TB during pregnancy in sub-Saharan Africa. We highlight the paucity of data on the effectiveness of integrated ANC models and identify significant structural barriers in the health system (funding, infrastructure, distribution, human resources), the adoption system (limited buy-in from implementers, leadership, governance) and, in the broader context, patient-centred barriers (fear, stigma, personal burdens) and barriers in funding structures. We highlight recommendations for action and discuss avenues for the global health community to develop systems to integrate multiple disease programs into ANC models of care that better address these three priority infectious diseases.

**Summary:**

With the current transition to the SDGs and concerns regarding the failure to meet maternal health MDGs, the global health community, researchers, implementers and funding bodies must work together to ensure the establishment of quality operational and implementation research to inform integrated ANC models. It is imperative that the global health community engages in a timely discussion about such implementation innovations and instigates appropriate actions to ensure advances in maternal health are sufficient to meet applicable SDGs.

## Background

The 2000 Millennium Development Goals (MDGs) established a global development framework aimed at improving health and saving the lives of the world’s poorest [1]. Three of the eight MDG targets related directly to health: reducing child mortality (goal 4); improving maternal health (goal 5); and combating HIV, malaria and other diseases (goal 6) (Table 1). The health outcomes described in these goals have overlapping risk exposures that lend themselves to common and integrated responses. Health remains a key focus of the Sustainable Development Goals (SDGs), which again encourage an integrated response to major global health issues [2].

**Table 1:** Millennium and Sustainable Development Goals addressing HIV, TB and malaria or maternal, newborn and child health and sub-Saharan African achievements and statistics

Millennium Development Goals [1]	2015 sub-Saharan Africa achievement [1]
Goal 4: Reduce Child Mortality	
Target 5: Reduce by two-thirds, between 1990 and 2015, the under-five mortality rate	52% reduction in child mortality
Goal 5: Improve Maternal Health	
Target 6: Reduce by three-quarters, between 1990 and 2015, the maternal mortality ratio	49% reduction in maternal mortality ratio
Goal 6: Combat HIV/AIDS, Malaria and Other Diseases	
Target 7: Have halted by 2015 and begun to reverse the spread of HIV/AIDS	51% reduction in new HIV infections
Target 8: Have halted by 2015 and begun to reverse the incidence of malaria and other major diseases	69% reduction in malaria mortality in the under-five age group
Sustainable Development Goals [2]	2015 sub-Saharan burden of disease
Goal 3: Good Health and Well-being	
3.1: By 2030, reduce the global maternal mortality ratio to less than 70 per 100,000 live births	546 per 100,000 live births [75]
3.2: By 2030, end preventable deaths of newborns and under-five children, with all countries aiming to reduce neonatal mortality to at least as low as 12 per 1000 live births and under-five mortality to at least as low as 25 per 1000 live births	Neonatal and under-five mortality is 29 and 83 per 1000 live births, respectively [75]
3.3: By 2030, end the epidemics of AIDS, TB, malaria and neglected tropical diseases and combat hepatitis, water-borne diseases and other communicable diseases	2.6 (0–20.1) new HIV infections per 1000 uninfected population^a^ [76] 281 (22–852) new TB cases per 100,000 population^a^ [76] 268.6 (3.6–460.9) new malaria cases per 1000 population at risk^a^ [76]
3.8: Achieve universal health coverage, including financial risk protection, access to quality essential healthcare services and access to safe, effective, quality and affordable essential medicines and vaccines for all	HIV treatment coverage 0–70%^b^ [77] TB treatment coverage 10–70%^b^ [77] IPTp coverage 17–40% [37] Use of ITNs 10–70%^b^ [77]

*ITN* insecticide-treated net, *TB* tuberculosis, *IPTp* intermittent preventive therapy in pregnancy

^a^2014 statistics for WHO Africa region

^b^2015 statistics for WHO Africa region

In the SDGs, combating HIV, tuberculosis (TB) and malaria now resides within a broader health goal – Ensure healthy lives and promote well-being for all at all ages (goal 3) – with a specific call to end the epidemics of HIV, TB and malaria by 2030 [2] (Table 1). Given the prominence of maternal, newborn and child health across multiple goals, classifying goals targeting the same beneficiaries as distinct and interdependent clusters has been suggested to enhance the achievement of the SDGs [3]. The World Health Organization (WHO) and other institutions have endorsed integrated antenatal care (ANC) as the best strategy to address the inter-related health issues of HIV, TB and malaria, as well as other vaccine-preventable infectious diseases and nutritional deficiencies, in the high risk groups of pregnant women and infants [4–8]. By providing a comprehensive disease prevention approach, integrated ANC services can help accelerate progress towards maternal, newborn and child health goals (MDG achievements and current burden of disease are outlined in Table 1) by offering an efficient mechanism to counter the impact of fragmented service delivery. An integrated ANC approach to these diseases seems highly plausible and intuitively beneficial. The global reach of ANC programs is extensive, with ANC coverage being approximately 70% in sub-Saharan Africa, the region that harbours the highest burden of HIV, malaria and TB globally and where ANC often represents a woman’s first contact with the healthcare system [4, 9]. Statistics on the potential impact of integrating these services into ANC remain limited, with no available published data on the impact of HIV services integrated into ANC on maternal morbidity or mortality, despite 25% of maternal deaths in sub-Saharan Africa being attributable to HIV [10]. Additionally, there is no data estimating the potential role of integrated ANC services on preventing mother-to-child HIV transmission, except for a recent systematic review (three of the four studies were based in sub-Saharan Africa) where a higher than two-fold increase was observed in pregnant women commencing antiretroviral therapy (ART) in ANC clinics integrating ART within their service compared to women attending clinics without ART integration [11]. A review of integrating intermittent preventive therapy in pregnancy (IPTp) and bed nets into ANC also estimated a reduction in all-cause child mortality of 32 and 18%, respectively [12]. Others have reported that incorporating TB screening and diagnosis into ANC may detect and treat an additional 167,200 TB cases globally [13], which would help reduce the 40% maternal mortality rate of untreated TB in pregnancy [14, 15]. In this context, integrated ANC offers enormous potential to reduce the global burden of disease through the provision of same day, same site testing and treatment for HIV/AIDS, malaria and TB as part of routine care for pregnant women.

Despite this potential, the rise in funding for single-disease programs, commonly focused on HIV/AIDS and malaria, undermines efforts to overcome fragmentation in health systems [16, 17]. Such a focus on single-diseases has extended to ANC; rather than focusing on integrated services that address multi-disease health priorities in maternal, newborn and child health, the literature largely describes integrated services that combine ANC with a single-disease focus (reviewed in [18]). We argue that, despite WHO recommendations, there is a remarkable lack of evidence on best practice approaches of program implementation for integrated ANC models in sub-Saharan Africa and beyond.

The issues for implementation of integrated ANC are broad and multifaceted. Here, we focus on implementation challenges of integrating HIV, malaria and TB services into ANC models in sub-Saharan Africa and identify a paucity of evidence demonstrating their effectiveness in addressing these multiple disease outcomes in these high risk populations. Our discussion of implementation challenges is guided by a conceptual framework that considers the problem – the prioritisation of SDGs in maternal, newborn and child morbidity and mortality and the complexity of overcoming implementation barriers – alongside the potentially effective intervention of HIV, TB and malaria services integration into ANC within the health system. We consider ways to improve the implementation of the integrated ANC intervention within a framework that first addresses health system strengthening to deliver quality service models through the development and maintenance of adequate physical and technical infrastructure. Second, we consider the adoption of the system and the need for buy-in from local governments and ANC staff to support integrated ANC models. Finally, we use the framework to consider the broader contextual factors such as the community and psycho-social impact of delivering prevention and care interventions, negative social attitudes to specific diseases, siloed and disease-specific funding models, and implementation and funding issues associated with the specific responsibilities assumed by different levels of government. We discuss avenues for the global health community to develop and maintain integrated ANC models of care, and consider how our findings are broadly relevant to other interventions aimed at mothers, their newborns and children. In 2016, with the transition from the MDGs to the SDGs and progress towards maternal, infant and child health goals lagging in sub-Saharan Africa (Table 1), it is imperative that the global health community discusses and instigates appropriate action to ensure sufficient advances in maternal, newborn and child health are made to accelerate the progress towards the relevant SDGs.

### Global guidelines for HIV, TB, malaria and ANC

The integration of HIV, TB and malaria interventions into ANC was given only limited attention in the WHO’s 2002 multicentre trial of a new evidence-based ANC model [19, 20]. Developed specifically for the management of low-risk pregnant women in resource-poor settings, the streamlined model of care that limited the number of tests and clinical procedures to those necessary for identifying special health conditions and women at risk of developing complications, performed similarly in randomised controlled trials with respect to maternal, newborn and child health outcomes (e.g. pre-eclampsia, urinary tract infections, low birth weight) to the standard “Western” ANC model [19, 20]. Beyond general recommendations for HIV rapid point-of-care testing and treatment initiation through specialised care referral for the prevention of mother-to-child transmission of HIV (PMTCT) and IPTp for malaria (prophylaxis in pregnancy in the absence of screening), the guidelines provided limited practical information on service implementation and disease screening and care.

The potential for integrated health services to address the three health-related MDGs received renewed attention in 2008 in the WHO’s technical brief, *Integrated Health Services – What and Why?* [16], where a renewed fight against health service fragmentation through integrated service delivery was called for. The technical brief outlined that, for the user, integrated healthcare should be “*seamless, smooth and easy to navigate*” ([16]; p. 5). Fully integrated ANC would see pregnant women in sub-Saharan Africa attending ANC clinics to receive health education, HIV counselling, testing and appropriate treatment, long-lasting insecticide-treated nets, IPTp, and TB screening and treatment (WHO guidelines summarised in Table 2) as well as other health services (e.g. screening and treatment of syphilis, anaemia and nutritional interventions) as required. In theory, such a model of integrated ANC should markedly improve the coverage, uptake and retention in care, and reduce the time to treatment initiation for these disease-specific health interventions. However, recent reports show that implementation of such service models in sub-Saharan Africa are minimal and the available data on HIV, malaria and TB services in ANC demonstrates that intervention coverage is low (Table 2).

**Table 2:** WHO guidelines, national guidelines and implementation coverage on HIV, malaria, TB, prevention, screening and treatment during pregnancy in sub-Saharan Africa

Intervention	WHO recommendations^a^	Inclusion in national guidelines^b^	Coverage (%)^c^; median (range)
HIV			
Prevention	ART prophylaxis from 14 weeks for PMTCT	Yes	No data
Screening	HIV testing at first ANC visit	Yes	37.9% (14.7–52.4%)
Treatment	ART for mother starting immediately if CD4 count ≤ 350 cells/mm^3^ for mother’s own health (treatment of HIV and PMTCT)	Sometimes	No data
Malaria			
Prevention	IPTp at each ANC visit from 2^nd^ trimester to delivery, doses ≥ 1 month apart, preferably DOT	Sometimes	6.6% (0.1–40.9%)^d^
	Receive one LLIN through ANC visits	Sometimes^e^	39.7% (16.0–75.4%)^f^
Screening	No guidelines	N/A	N/A
Treatment	‘Effective case management’ – presumptive treatment of symptoms/fever as per national guidelines	Rarely	No data
TB			
Prevention	TB infection control in PMTCT settings	No	No data
Screening	TB signs and/or symptoms should be evaluated for active TB HIV positive pregnant women should be screened for TB during HIV post-test counselling	Rarely Rarely	No data No data
Treatment	All first-line TB drugs (except streptomycin)	No	No data

*ANC* antenatal care, *ART* antiretroviral therapy, *DOT* directly observed treatment, *IPTp* intermittent preventive therapy in pregnancy, *ITN* insecticide-treated net, *LLIN* long-lasting insecticide-treated net, *SP* sulfadoxine-pyrimethamine, *TB* tuberculosis, *PMTCT* prevention of mother-to-child transmission

^a^Adapted from WHO guidelines [78–85]

^b^National Guidelines of the nine countries (Botswana, Tanzania, Uganda, Liberia, Mozambique, Namibia, Nigeria, South Africa, and Swaziland) with accessible guidelines/policies/strategies on Ministry of Health (or similar) websites. The recommended components of ANC vary widely between and within countries, often depending on the level of care (community, district, regional or national) at which ANC is provided. Other components generally included in ANC are blood tests for blood group, anaemia, HIV and syphilis, urine dipstick for protein and glucose, checking blood pressure, providing tetanus toxoid, iron and folic acid, assessment of substance use, and performing nutrition and hygiene counselling

^c^Implementation coverage data is based on STATcompiler: The DHS Program. Data is median (range) coverage data available for HIV screening (data from four countries) and malaria prevention (IPTp 34 countries; ITN/LLIN 25 countries)

^d^Coverage is reported as percentage of women receiving 3+ doses of SP/Fansidar, with at least one dose during ANC visit (DOT)

^e^Including any reference to provision of LLINs, ITNs or mosquito nets in general

^f^Percentage coverage of pregnant women who slept under a LLIN the night before the survey

To date, published research into the effectiveness of integrated ANC in sub-Saharan Africa has almost exclusively focused on HIV and malaria. Controlled trials and observational studies generally demonstrated relative increases in pregnant women and infants being tested for HIV (range 40–200%), initiating treatment for the PMTCT (35–400% relative increase) and reductions in loss-to-follow-up (50% relative reduction) within integrated versus non-ANC-integrated approaches across a number of sub-Saharan African countries [11, 18, 21–33]. Integration of HIV into ANC is likely to have contributed significantly to achieving an ART coverage to prevent PMTCT of 73% among pregnant women living with HIV globally [34]. While this increase in coverage will play a role in helping achieve global 90-90-90 HIV prevention targets (90% diagnosed, 90% on treatment and 90% virally suppressed [35]) and are key to PMTCT, men remain the primary target for the HIV prevention impact achieved from increasing ART coverage in sub-Saharan Africa, given they are the dominant source of transmission [36]. Incorporation of IPTp for malaria into ANC in sub-Saharan Africa has been recommended for over a decade, but calls for IPTp integration have achieved more moderate success, with only 40 and 17% of women living in malaria endemic areas receiving more than two or three doses of IPTp, respectively, in 2015 [37]. Compared with HIV and malaria, there is a paucity of data on the effectiveness of integrating TB care into ANC clinics. A pilot study in Kenya, examining the integration of TB screening and treatment into ANC, reported a 91% increase in the number of women screened for TB; however, no outcomes on treatment adherence or clearance of infection were reported [38].

Given the call for multiple-disease, fully integrated ANC models [16], it is disappointing that nearly all the published literature focuses on the integration of single-disease services into ANC. This represents a crucial gap and impedes the adoption of evidence-based approaches in the development of effective integrated ANC services that target multiple diseases. The identification of the barriers and enablers for the successful integration of services that simultaneously address multiple diseases is needed to optimise new ANC models and vitally important to support progress towards meeting global development goals related to maternal, newborn and child health.

### Challenges in implementing integrated ANC: structural barriers in health infrastructure and adoption systems

Many challenges exist in cross-disease integrated ANC in sub-Saharan Africa. Structural barriers often manifest in the characteristics of the health system (Table 3). Weak program links, health systems, and financial infrastructure are cited as a prominent barriers to ANC attendance and integrated ANC service delivery (systematically reviewed in [18, 39, 40]). For example, the provision of HIV treatment at specialised ART clinics separate to ANC clinics reduced uptake of HIV services in pregnant women by around 50% [21, 29, 30]. To counter this problem, governments, in conjunction with non-governmental organisations, have begun integrating HIV services and PMTCT into primary healthcare and ANC with the recognition that HIV-specific resources could be allocated diagonally (aiming for disease-specific results through enhanced health systems) [18, 41]. However, integration of these services can be hindered if not accompanied by sufficient additional funding to ensure cohesive and effective service integration; limited resources remain a major barrier in the integration of malaria treatment and prevention into ANC [42]. To achieve effective integration of HIV, TB and malaria interventions into ANC services, implementation must be accompanied by improved physical and technical infrastructure; services provided at different locations (e.g. consulting rooms and laboratories in different facilities) or those not provided on the same day pose a significant challenge to integrated HIV services (reviewed in [11, 26, 43]) and have impacted on the integration of malaria treatment into ANC [44, 45]. While IPTp is given presumptively in ANC, thus removing the need for testing, women are often required to purchase the drug elsewhere or are not directly observed taking therapy having been given the drug to take home, both of which have been identified as a significant barrier to IPTp coverage [45–47]. Effective procurement and distribution systems are also important determinants of success for integrated services, with inadequate and irregular supplies of essential drugs and interventions, in both the public and private healthcare sector, representing a major barrier in the uptake of both integrated HIV and malaria services [18, 39, 40].

**Table 3:** Key health system, adoption system and broader context barriers to integration of HIV, TB and malaria services into antenatal care

Health system characteristics	
Insufficient resources and financial infrastructure	• Weak disease-specific and antenatal care program links
	• Weak health systems
	• Financing of health services may be required to change to fit integration of previously separate services
Inadequate physical and technical infrastructure	• Services provided at different locations
	• Services not provided on same day
Ineffective procurement and distribution systems	• Patient drug procurement/administration at different locations
	• Inadequate and irregular supplies of essential drugs and interventions
Inadequate information systems	• Weak monitoring and evaluation systems
Insufficient human resources	• Staff shortages and overburdened staff
	• Frequently reallocated workforce
Adoption system	
Insufficient buy-in from healthcare implementers	Buy in can be affected by:
	• Limited human resource capacity, time, training and financing for extra
	• Service delivery tasks
	• Lack of supervision
	• Poor motivation
Inconsistent leadership and governance	• Inconsistent national policies
	• Inconsistent guidelines and training documents
	• Poor adherence to guidelines
Broader context	
Patient-centred barriers to service delivery	• Attending multiple clinics on separate occasions/locations
	• Time away from work/parenting obligations
	• Costly and timely transport options
	• Lack of partner support
Cultural and social barriers	• Fear and stigma, lack of trust in interventions
	• Societal attitudes towards HIV, tuberculosis, malaria
Funding structures	• Historical focus on donor funding to specific diseases
	• Siloed and disease-specific funding models
	• Complexities of different levels of government funding

The level of buy-in from staff and the capacity of service infrastructure to support the adoption of new service delivery models also represent key targets to overcome structural barriers for integrated ANC; here, adoptions systems influence the human resource characteristics of the health system (Table 3). Integrating service delivery of multiple interventions into ANC in resource-constrained settings such as sub-Saharan Africa can significantly impact the health workforce. Staff shortages, overburdened staff and a frequently re-allocated workforce have limited the success of HIV, TB and malaria service integration into ANC [4, 11, 18, 39, 40, 44, 47–55]. Together with unsupervised healthcare staff and a lack of adequately trained staff, these human resource constraints will affect motivation and buy-in from those charged with implementing integrated ANC. Furthermore, a lack of leadership and governance can compound the adoption of new health systems. For example, a study assessing the consistency of national-level policies and guidelines for malaria in pregnancy in five sub-Saharan Africa countries found major inconsistencies both within and between countries in national policy and training documents [56]. Poor adherence to guidelines [40], coupled with outdated and inconsistent guidelines, may lead to incorrect implementation of prevention and care strategies and low coverage of malaria, HIV and TB interventions into ANC.

By identifying common structural barriers to implementing integrated ANC in the health and adoption systems, enablers can also be identified to optimise service delivery and improve maternal and child health outcomes*.* Enablers that have helped overcome identified structural barriers in integrated ANC, reproductive health and primary care services include streamlined funding, effective distribution/logistical systems, integrated health information systems [21, 30, 33, 44], provision of all services on the same site [21, 57], free or low-cost testing and treatment [57], holistic, comprehensive healthcare delivered by multi-skilled health workers or different health workers under the same roof [57], provision of support (psychosocial, financial) to encourage treatment adherence and clinic attendance [57–59], and well-supported trained healthcare staff [33, 42, 49, 60–63]. Careful consideration of these enablers in the design and implementation of integrated ANC, HIV, TB and malaria services is required and will generate demand for such integrated services. It is also imperative to have appropriate process, outcome and impact evaluations that result in fundamental translatable outcomes for integrated ANC, HIV, TB and malaria services. Rigorous evaluations of the integration of multiple interventions into ANC are important given the integration of approaches to tackle multiple diseases may exponentially amplify implementation issues in the absence of appropriate infrastructure or human resource strengthening.

### Challenges in implementing integrated ANC in the broader context: patient- and community-centred barriers

In the broader context, the acceptability of models of health service delivery from a community and individual patient perspective can significantly impact the reach of health services and their effectiveness in achieving improved health outcomes (Table 3). Integrated ANC has the potential to either reduce or exacerbate (particularly for stigmatised diseases), patient-level barriers to care. Studies conducted in sub-Saharan Africa focusing on the integration of HIV testing and treatments into ANC clinics and IPTp access at ANC have cited the need to attend health clinics on multiple occasions and at separate locations, taking time away from work or parenting obligations, and costly and timely transport options as major barriers to returning to health clinics [11, 21, 30, 33, 64–68]. These access issues were relevant to receiving HIV test results and initiating and adhering to HIV treatment and PMTCT services [21, 29, 30, 33, 62, 64], and similar problems were identified with TB services and the need to return to clinics to provide a second sputum to confirm a diagnosis [65]. These considerations support the principles underpinning integrated ANC models by underscoring the importance of streamlining service provision across multiple antenatal and disease-specific needs.

While the convenience of an integrated approach appears favourable to the patient, the broader contexts in which different diseases are perceived within communities, including their social acceptability and potential disease-related stigmas, give rise to challenges for integrated ANC. In the case of malaria, a systematic review found that the general lack of awareness among women of why they were being given IPTp, as well as its safety, regimen or benefit, was a key barrier to the implementation of IPTp into ANC services [40, 69]. Illness, shyness, low social position and lack of partner support were also identified as social barriers that prevented or delayed ANC attendance and IPTp adherence [40, 70–72]. Studies investigating social barriers to accessing HIV care in rural Kenya found that 82% of HIV-infected women preferred integrated HIV ANC services, with service satisfaction among HIV-negative women relatively unaffected by the provision of HIV care [64]. It was suggested that high levels of satisfaction among HIV negative women was potentially due to the limited distinction in observed care provided to women on the basis of their HIV status [64]. However, the potential remains for integrated HIV services to act as a barrier to ANC attendance. Data collected from a District Hospital in Western Kenya showed that pregnant women were anxious they would be tested for HIV without consent if they attended ANC clinics, with such concerns potentially hampering efforts to provide other health interventions through ANC clinics such as IPTp or insecticide-treated nets for malaria prevention [73]. Other studies identified the stigma and co-occurrence of HIV with TB as a potential barrier to integrated ANC service provision. Data collected from ANC clients and service providers in Malawi found stigma attached to TB was attributed to both fear of spread of infection and its association with HIV [65, 74]. Patients reported that ‘new’ TB was often perceived as being linked with HIV and expressed concern that TB patients were being viewed as AIDS patients [65]. Cultural and social barriers to healthcare, such as those associated with stigma, fear of diagnosis and a lack of trust, highlight the need for operational and implementation research to explore optimal ways to reduce these barriers.

### Concluding remarks in the broader context of funding structures

Despite the long-standing endorsement of integrated ANC services to improve health outcomes among women and children at risk of HIV, TB and malaria, there is little literature to guide the implementation, refinement and evaluation of such services. There may be additional grey literature that describes successful service integration and it is imperative to develop avenues to consolidate this literature and make it accessible to researchers, program implementers and policymakers. The highest burden of these diseases is in sub-Saharan Africa, and what little published research exists on integrated ANC is focused there. However, the considerable variation in healthcare and the economic, social and political context in sub-Saharan Africa warrants further research on integrated ANC models representing a greater breadth of African countries. Studies outside of the African continent are also crucial to inform local models given the distinctive regional considerations, including those related to health systems, geography, the relative involvement of government, aid agencies and civil society in the provision of health services, and the social, cultural and legislative positions on specific diseases and their routes of transmission.

With the current transition to the SDGs and concerns centred around progress towards MDGs related to maternal, newborn and child health [9], the global health community, researchers, implementers and funding bodies must work together to ensure the establishment of quality operational and implementation research into models of integrated ANC that address multiple health outcomes. This is particularly pertinent because, while our essay concentrated on HIV, TB and malaria, other communicable and vaccine-preventable diseases (e.g. viral hepatitis) also feature in the new SDGs (end epidemics by 2030, Table 1) [2] alongside integration of reproductive health into national strategies [2], and have the potential to be incorporated into a program of integrated ANC. Recent analysis of disbursements of development assistance for health show that, following a decade of increased funds for the MDG-related diseases of HIV, TB and malaria, since 2010, funding for these diseases has largely flat-lined while funds for maternal, child and newborn health have increased [17]. The broader context of these changes in decision-making about global health funding priorities (Table 3) underscore a move away from single-disease funding allocations and an opportunity for significant progress towards improved maternal health outcomes through the SDGs. However, given the significant burden of disease that HIV, TB and malaria represent for young women and their children, integrated ANC is an increasingly important model to maintain progress on disease-specific prevention while simultaneously addressing shortfalls in maternal and child health outcomes. To achieve a successful transition to the SDGs and guide best practice approaches when international donor funding is static or predicted to decline, monitoring and evaluation systems need to accompany program implementation to determine the effectiveness of integrated models of ANC in improving health outcomes (Box 1). Increasing and sustaining investment in ANC is a predictable barrier to implementing and maintaining integrated ANC. It is therefore imperative, in the current economic climate, that practical and resource-efficient solutions to improving population-level health outcomes are found. With maternal, newborn and child morbidity and mortality remaining unacceptably high in resource-constrained countries, especially in sub-Saharan Africa, the need for expanded operational research aiming to identify smarter solutions to improving maternal health is critical and requires strong leadership from the global health community.

## Box 1: Recommendations for action

At the macro-level:

 • Global funding bodies ensure their monitoring and evaluation outcomes are aligned with the implementation and operational research outcomes of program implementers and researchers

 • Global funding bodies make sufficient funding available to enable this data collection and analysis to occur as part of routine monitoring and evaluation

 • Research institutes and global funding bodies work towards expanding global mentoring programs that provide training, guidance and support to implementers and researchers in low- and middle-income countries

 • Program implementers and researchers work collaboratively to produce outcome-based research that results in academic publications as a form of dissemination of findings and in changes to policy and service delivery programs

At the implementation level:

 • Streamlined funding, distribution/logistical systems, monitoring and evaluation systems

 • Prioritise holistic, comprehensive healthcare

 ○ Ensuring adequate resources and staff training, promote the development of a fluid multi-skilled workforce

 ○ Increase physical and technical infrastructure and streamlining the provision of services to reduce attendance time and loss to follow-up

  ▪ Promote processes to facilitate same day provision of multiple clinical and preventive services

  ▪ Integration of consulting rooms with laboratory services

  ▪ Investment into point-of-care tests to alleviate the need for laboratories

 • Provision of testing and treatment for free or at a low cost

 • Provision of psychosocial support in encouraging treatment adherence and clinic attendance

 • Ensuring similar levels of perceived antenatal care in disease-affected and unaffected women

 • Increasing trust and consumer confidence in therapeutic efficacy of drugs and transparency around HIV testing

 • Public education and health promotion strategies to increase disease awareness and trust in antenatal care intervention strategies

Research priorities:

 • Determine the effectiveness of integrating tuberculosis care into antenatal clinics

 • Monitor the therapeutic efficacy of treatment and prophylaxis in an era of emerging drug resistance

 • Quantify the impact of enablers to reduce structural and patient-centred barriers on the uptake of HIV, tuberculosis and malaria services

 • Quantify the impact of stand-alone versus cross-disease models of care

## Abbreviations

AIDS: Acquired immunodeficiency syndrome
ANC: Antenatal care
HIV: Human immunodeficiency virus
IPTp: Intermittent preventive therapy of malaria in pregnancy
MDGs: Millennium development goals
PMTCT: Prevention of mother-to-child transmission
SDGs: Sustainable development goals
TB: Tuberculosis
WHO: World Health Organization
